# Clinical and economic burden of invasive fungal diseases in Europe: focus on pre-emptive and empirical treatment of *Aspergillus* and *Candida* species

**DOI:** 10.1007/s10096-013-1944-3

**Published:** 2013-09-12

**Authors:** L. Drgona, A. Khachatryan, J. Stephens, C. Charbonneau, M. Kantecki, S. Haider, R. Barnes

**Affiliations:** 1 Department of Haematology/Oncology, National Cancer Institute and Comenius University, Bratislava, Slovakia; 2 Pharmerit International, 4350 East West Highway, Suite 430, Bethesda, MD 20814 USA; 3 Pfizer Global Outcomes Research, Pfizer Inc., New York, NY USA; 4 Pfizer International Operations, Pfizer Inc., Paris, France; 5 Pfizer Global Research and Development, Pfizer Inc., Groton, CT USA; 6 Cardiff University School of Medicine, Cardiff, UK

## Abstract

Invasive fungal diseases (IFDs) have been widely studied in recent years, largely because of the increasing population at risk. *Aspergillus* and *Candida* species remain the most common causes of IFDs, but other fungi are emerging. The early and accurate diagnosis of IFD is critical to outcome and the optimisation of treatment. Rapid diagnostic methods and new antifungal therapies have advanced disease management in recent years. Strategies for the prevention and treatment of IFDs include prophylaxis, and empirical and pre-emptive therapy. Here, we review the available primary literature on the clinical and economic burden of IFDs in Europe from 2000 to early 2011, with a focus on the value and outcomes of different approaches.

**Electronic supplementary material:**

The online version of this article (doi:10.1007/s10096-013-1944-3) contains supplementary material, which is available to authorized users.

## Introduction

Opportunistic invasive fungal diseases (IFDs) are a significant cause of morbidity and mortality in immunocompromised patients, and are associated with increased healthcare costs [1].

Early diagnostics can improve treatment outcomes and potentially reduce IFD-associated costs. Microscopy and histology are practical and inexpensive methods for IFD detection, but cannot identify organisms to the species level and are often invasive. Non-invasive methods include techniques to detect fungal antigens (e.g. galactomannan, mannan, β-D-glucan) or fungus-specific nucleic acids [polymerase chain reaction (PCR)], or rely on specific radiological and clinical signs. IFDs can be classified as possible, probable or proven based on host, clinical and microbiological features using the European Organization for Research and Treatment of Cancer/Mycoses Study Group (EORTC/MSG) criteria [2].

**Fig. 1:**
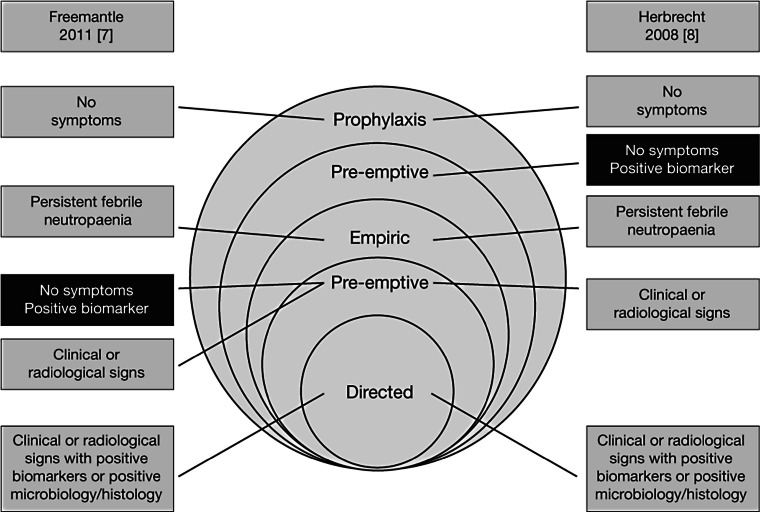
Variable definitions of treatment strategies and relative positions within the treatment continuum

In addition to the antifungal treatment of confirmed diseases, approaches include prophylaxis, empirical therapy and pre-emptive/diagnostic-driven therapy. Prophylaxis is recommended for the prevention of infection in high-risk patients [3–6]. Empirical therapy is an early approach in patients with persistently febrile neutropaenia unresponsive to antibiotic therapy. Pre-emptive/diagnostic-driven therapy is usually based on the presence of specific clinical signs and fungal biomarkers, but there is no consensus on the definition and there may be overlap with empirical and targeted therapy (Fig. 1) [7, 8].

Polyenes, azoles and echinocandins are used to treat IFDs. These agents demonstrate high levels of antifungal activity, although resistance is reported for all classes. Antifungal treatment can be hampered by toxicity, poor tolerability or a narrow activity spectrum, and limitations have driven efforts to determine the efficacy of combination therapy, but no optimal treatment strategy has been identified [9]. The Infectious Diseases Society of America (IDSA) currently recommends fluconazole or an echinocandin as the first-line therapy for candidiasis in non-neutropaenic patients; for neutropaenic patients, initial therapy with echinocandins is preferred until the *Candida* species is identified [10]. Voriconazole is the recommended therapy for invasive aspergillosis (IA) [11–13].

The aim of this article is to review the primary evidence on the clinical and economic burden of IFDs in Europe, encompassing the value and treatment outcomes of different diagnostic and therapeutic approaches to management.

## Methods

A strategic literature review was conducted to understand the clinical burden of IFD in terms of epidemiology, outcomes and treatment trends, and the economic burden of IFD contributing to overall healthcare resource utilisation (HCRU), including hospitalisations, length of stay (LOS) in hospital, diagnostic procedures and additional treatment and medications.

### Literature search

PubMed/MEDLINE and EMBASE were searched to identify the primary literature on IFDs (Supplementary Table 1). Searches were limited to results published in English; during the last 10 years (2000 to early 2011); and having reported on human subjects. Abstracts from clinical congresses [International Society for Pharmacoeconomics and Outcomes Research (ISPOR), Interscience Conference on Antimicrobial Agents and Chemotherapy (ICAAC), IDSA and the European Society of Clinical Microbiology and Infectious Diseases (ECCMID)] were searched for data that may still have been in press. Abstract searches were limited to results published in English; during the last 5 years (2005 to 2010); and having reported on human subjects. Key review papers and reports issued by disease surveillance agencies were utilised to define targeted searches.

#### Study eligibility and selection criteria

Available abstracts, full-text articles and other materials were reviewed for inclusion using the criteria in Supplementary Table 2.

## Results

### Literature analysis

**Fig. 2:**
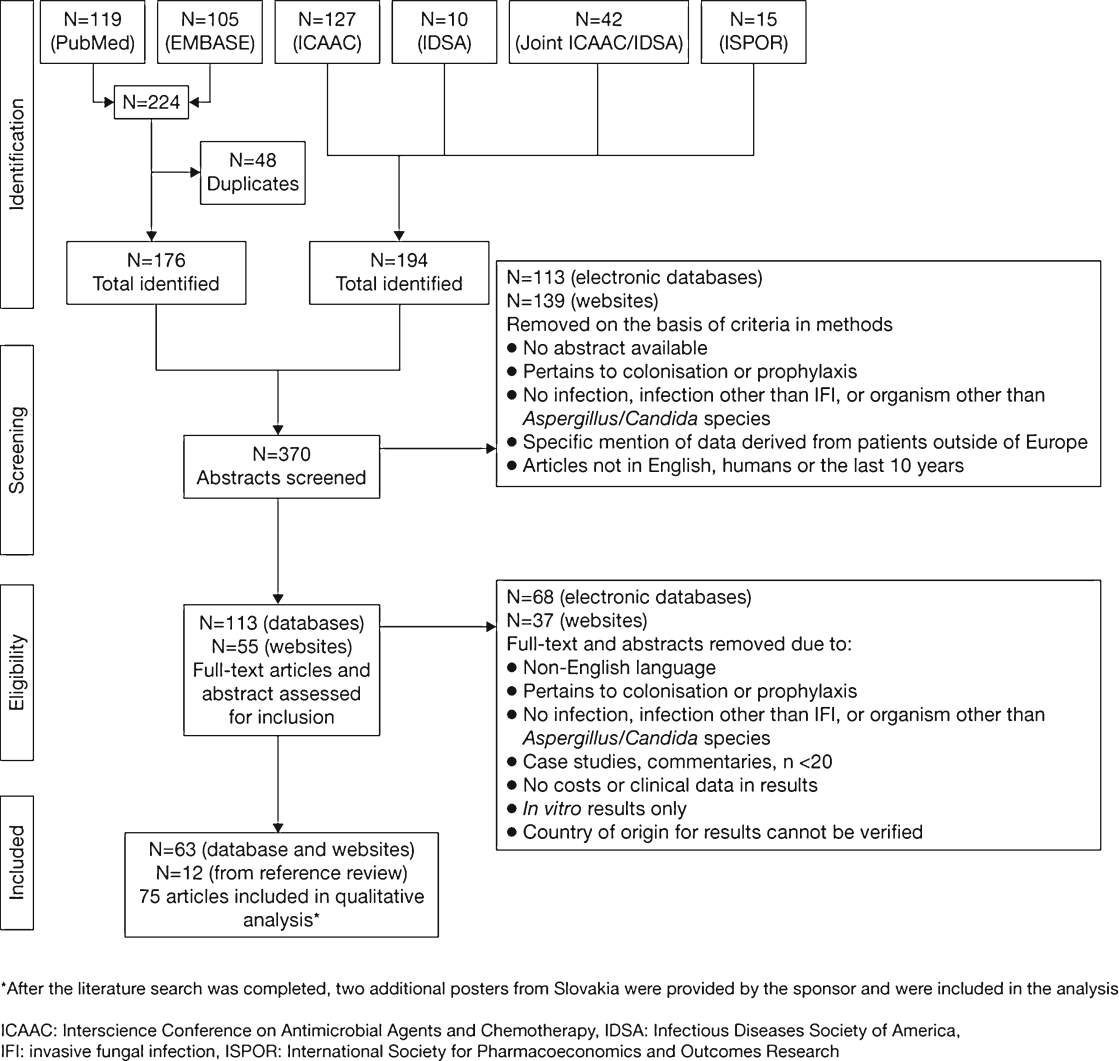
Flow diagram for literature identification

In total, 224 primary literature articles and 194 clinical abstracts were identified. After eligibility criteria were applied, 113 primary literature articles and 55 clinical abstracts were reviewed. Bibliographies were screened, which identified 12 primary literature articles. The final literature field consisted of 57 primary literature articles and 18 clinical abstracts (Fig. 2) [14–88]. Two additional posters from Slovakia were later provided by the sponsor and included in the analysis [89, 90].

### Clinical burden: mortality

#### IFD-related mortality

Twenty-one studies were identified across ten countries, including one European-wide study, which explicitly reported IFD-related or attributable mortality. However, a clear definition of IFD-related death was lacking in many studies, with criteria ranging from all deaths during the study to patients with hyphal invasion on autopsy. Many patients had underlying conditions; differentiating mortality related to these conditions from deaths directly caused by IFD was problematic. Furthermore, the proportion of IFD-attributable deaths was calculated using a variety of methods (e.g. in relation to the total number of deaths or in relation to the total population). Supplementary Table 3 reports the rates of IFD-related mortality and risk factors by European country.

#### Overall mortality

The overall 28- or 30-day mortality burden in patients with IFDs ranged from 1.5 to 82.4 %, with most estimates falling between 35 and 45 %. Three studies fell outside this range, with mortality estimates of <30 and >45 % [20, 33, 39]. These mortality rates may be explained by specific patient populations, with the highest 28-day mortality rates (66.7–82.4 %) in patients in an intensive care unit (ICU) with probable or proven IA [20].

The 12-week overall mortality ranged from 22 to 47 % [15, 19, 33, 51, 61]. Other time frames for mortality measures were 7, 14, 42, 60 and 100 days, 4 months and 4 years, and mortality estimates ranged from 5 % in haematopoietic stem cell transplant (HSCT) patients (at 100 days) [82] to 70.6 % (at 14 days) in an ICU population with IA [20]. Twenty-one studies reported mortality over an unspecified time frame. The highest overall mortality rate (90 %) was reported in paediatric patients with haematological malignancies and IA [76], and in ICU patients with *Candida* non-*albicans* infection [45].

The overall mortality due to *Candida* infection ranged from 20 to 90 %; however, after the exclusion of ICU populations, this narrowed to 20–32 %. In a study by Almirante and colleagues, the rates of overall mortality of 23 and 43 % were reported for patients with *C*. *parapsilosis* and *C*. *albicans*, respectively (*p* = 0.003) [64].

The overall mortality range for *Aspergillus* infections was 30–90 %, and remained high, irrespective of whether known ICU populations were excluded.

These results indicate that mortality rates in patients receiving antifungal treatment depend on the time period over which mortality is measured, the responsible organism and the underlying disease characteristics of the patient.

### Diagnosis of IFD

#### Antigen-based assays

Galactomannan testing for the diagnosis of *Aspergillus* infections was reported in 20 studies across ten countries. All of the studies used serum specimens, and three also used bronchoalveolar lavage (BAL) fluid [15, 22, 61]. BAL testing was preferred in the study by Slobbe and colleagues [61], and serum was only used when a bronchoscopy could not be performed. The testing frequency ranged from twice weekly [28, 78, 79] to daily monitoring [17] as part of a screening strategy. Four studies measured galactomannan following a triggering event as part of an intensive diagnostic workup (IDWU) [18, 51, 57, 61]. Optical density cut-offs ranged from >0.5 to ≥1.5; the most frequently used criteria for positivity was an optical density index ≥0.5 for two consecutive tests.

Reports varied regarding the utility of galactomannan testing in the diagnosis of IFD. Barnes and colleagues investigated the use of a febrile neutropaenia care pathway that used routine biomarker testing in patients with acute leukaemia, refractory disease undergoing aggressive chemotherapy or undergoing HSCT [79]. In this study, 61 patients were positive for *Aspergillus* by PCR; of these, 25 (41 %) were positive using the galactomannan assay. Thirty-two patients were positive by galactomannan testing and all but seven were also positive by PCR. Thirty-six patients were positive by *Aspergillus* PCR alone. Antigen testing (galactomannan and mannan) was less sensitive than PCR but demonstrated good specificity. Combined antigen and PCR testing gave a sensitivity of 100 and 87.5 % for single and multiple results, respectively, and a specificity of 100 %.

Girmenia and colleagues used galactomannan testing as part of an IDWU and reported detection in 89 % of cases [51]. Galactomannan assays contributed to diagnosis in 26 of 100 IDWU as a result of at least two positive samples, while 42 of 100 IDWU computed tomography (CT) findings led to IFD diagnosis. Both galactomannan tests and high-resolution CT (HRCT) scans were positive in the same IDWU in 74 % of patients who were diagnosed with pulmonary IFD. The detection of galactomannan occurred after a mean of 4.7 days (range 3–7 days) following the CT results in four cases, and galactomannan test results were negative despite positive culture findings in three cases [51]. No further data regarding the specificity or sensitivity were provided.

Mannan testing for the diagnosis of *Candida* infections was reported in two studies, each using a minimum cut-off of 0.5 ng/ml [56, 79]. In the aforementioned study by Barnes and colleagues [79], testing was performed twice weekly in conjunction with other biomarker tests. Eleven patients had positive *Candida* PCR results, five of whom also had a positive mannan antigen assay.

Posteraro and colleagues reported success with the use of *Candida* mannan screening versus *Candida* culture surveillance in BAL in the neonatal ICU [56]. Sixteen infants with positive mannan assays were compared with 16 historical controls with positive surveillance cultures. The incidence of IFD in the surveillance culture group was 23 %; no cases of IFD were reported in the group undergoing mannan screening.

β-D-glucan is not specific for *Aspergillus* species and was only referred to in a nationwide *Aspergillus* registry in Austria, in which (1,3)β-D-glucan testing was used in 3 % of cases as a diagnostic tool [15].

#### PCR-based testing

Studies conducted in the UK and Austria reported that PCR testing was routinely performed in 4 and 16 % of cases, respectively [15, 41]. PCR assays were used in a variety of ways in addition to screening/pre-emptive strategies. For example, in a study conducted in Germany, a positive PCR assay of BAL specimens was used in conjunction with suspicious CT findings for infection diagnosis [38]. By contrast, Rubio and colleagues performed PCR testing as a confirmation assay in patients who were positive for invasive filamentous fungal infection by pathology [76].

A prospective study conducted in Germany compared outcomes in patients following allogeneic HSCT who were treated empirically with antifungals versus those treated pre-emptively after PCR testing [39]. Patients treated with antifungals after a positive PCR assay had significantly decreased mortality at 30 days (*p* = 0.015), but this survival benefit did not persist at 100 days. Therefore, while pre-emptive treatment following a positive PCR assay appears to confer survival benefits, routine use in the clinical setting may present challenges [39].

Barnes and colleagues reported on the reproducibility and rapidity of PCR test results versus antigen testing by performing assays for both *Aspergillus* and *Candida*. All cases of proven disease were positive by both PCR and antigen testing [79]. A comparison of the PCR and galactomannan results revealed simultaneous positive results in six patients, a positive PCR prior to galactomannan testing in 15 patients and a positive galactomannan test prior to PCR in four patients [79].

#### HRCT

Most studies that reported use of HRCT were conducted in oncology patients; few details were provided regarding reasons for performing an HRCT scan (e.g. clinical suspicion or as part of routine monitoring). Weisser and colleagues reported on the use of HRCT scans in 161 episodes of infection in 107 patients with haematological malignancies [78]. Scans were performed once weekly or when clinically indicated. The halo sign, air crescent sign or cavitatory lesions were classified as major signs from HRCT scans, whereas all other infiltrates were classified as minor signs. Minor signs were reported in 43 % of cases and major signs in 7 % of cases; no infiltrate was seen with 50 % of the infection episodes.

Two studies reported the use of diagnostic HRCT scans in populations other than oncology patients. In one study, a suggestive HRCT was noted in 41 % of patients in a general hospital who had a positive pulmonary isolate for *Aspergillus* [63]. In the second study, which focused on critically ill patients with neutropaenia, an HRCT scan was performed in 17/67 patients with probable or proven IA with abnormalities on chest X-ray. Of these, three patients had a halo sign and nine had cavitation. The remaining five patients had non-specific changes [17].

### Early management approaches

#### Prophylaxis

The use of antifungal prophylaxis ranged from 28 to 100 % in 21 identified studies across 12 countries. All azole- and amphotericin-type products were used, and fluconazole was the most frequently reported prophylactic agent. Most studies did not address breakthrough infections, although a rate of 30 % was reported in an Austrian registry that defined breakthrough infection as proven fungal infection after 7 days of prophylaxis [15]. Therapeutic drug monitoring was not performed in this study; therefore, low drug concentrations could not be excluded as the reason for breakthrough infection.

#### Empirical therapy

Lafaurie and colleagues conducted a prospective chart review of empirical therapy in a heterogeneous population of patients admitted to haematology, oncology, ICU or infectious-disease wards in a French hospital [31]. Empirical treatment was initiated for refractory fever (39 %), recurrent fever (48 %), clinical signs of sepsis (5 %) and tachycardia with elevated C-reactive protein (CRP) (4 %) [31]. Caspofungin was the first-line treatment in 71 % of episodes, followed by liposomal amphotericin B (18 %) and amphotericin B deoxycholate (11 %) [31]. Eleven percent of patients had breakthrough infection (any IFD identified after 3 days of empirical treatment), although this could be attributed to the high-risk population.

In a second study focusing on empirical therapy, a retrospective chart review of patients undergoing induction of salvage chemotherapy for leukaemia given antifungal therapy on day 4 of fever with negative cultures was conducted [57]. The fungal-attributable mortality was 2.5 %, leading the authors to conclude that, while empirical therapy has become a controversial treatment modality, it may be of value in selected high-risk populations [57].

#### Pre-emptive therapy

Eight identified studies reported some form of a pre-emptive strategy, although definitions were diverse. Criteria for treatment initiation and the specific approaches used were different for all pre-emptive studies (Table 1). The presence of clinical signs and symptoms were triggers for treatment initiation in two studies [28, 79]; HRCT scans alone or with clinical microbiological or positive galactomannan tests triggered treatment in five studies [17, 51, 74, 79, 82]; galactomannan testing was used in four studies [17, 25, 51, 79], with one study using the *Candida* mannan assay [79]; PCR testing was used in two studies [39, 79]. A variety of treatment regimens were given to patients. Seven studies pre-defined drug treatment, while one study did not [51].

**Table 1:** Diagnostic methods in pre-emptive treatment

		Pre-emptive group criteria				
Reference	Country	Clinical	HRCT	GM/M	Microbiological	PCR
Non-comparative studies						
Maertens et al., 2005 [17]	Belgium		X	X	X	
Girmenia et al., 2010 [51]	Italy		X	X	X	
Posteraro et al., 2010 [56]	Italy			X		
Aguilar-Guisado et al., 2010 [74]	Spain	X	X		X	
Barnes et al., 2009 [79]	UK	X	X	X	X	X
Dignan et al., 2009 [82]	UK		X			
Randomised, comparative studies						
Cordonnier et al., 2009 [25]	France	X	X	X		
Hebart et al., 2009 [39]	Germany					X

*GM* galactomannan, *HRCT* high-resolution computed tomography, *M* mannan, *PCR* polymerase chain reaction, *UK* United Kingdom

Overall, pre-emptive treatment was initiated in 7.7–51.7 % of patients [17, 25, 39, 51, 74, 79, 82]. Most studies reported pre-emptive antifungal use of between 39.2 and 51.7 %. Three studies reported low rates of pre-emptive treatment initiation [17, 51, 82]. Maertens and colleagues had the lowest number of patients receiving therapy due to strict requirements for treatment initiation (two positive galactomannan assays or positive microbiological results with suggestive CT findings) [17]. Dignan and colleagues had strict definitions for antifungal treatment initiation, which resulted in 17 % of patients receiving pre-emptive treatment [82]. Caspofungin was started: if there was a positive CT and neutropaenic fever; if a CT could not be performed within 24 h; or at the discretion of the physician in patients who developed respiratory failure with high dependency or were in the ICU [82]. In another study, only one patient in the pre-emptive therapy arm received treatment secondary to a negative IDWU and worsening clinical condition [51].

These findings indicate a lack of consensus and understanding of the definition of pre-emptive therapy. Therefore, the applicability of currently available data to the wider clinical setting is unclear.

#### Empirical versus pre-emptive strategies

Ten studies across six countries reported on some form of empirical or pre-emptive therapy. A summary of non-randomised, observational studies of empirical and pre-emptive treatments in adult patients is presented in Table 2. Two prospective randomised studies conducted in oncology patients directly compared empirical and pre-emptive strategies (Table 3) [25, 39].

**Table 2:** Summary of non-randomised, observational studies of empirical and pre-emptive treatments in adult patients

Country	Belgium (Maertens et al. 2005) [17]		Italy (Girmenia et al. 2010) [51]		Spain (Aguilar-Guisado et al. 2010) [74]		UK (Dignan et al. 2009) [82]		UK (Barnes et al. 2009) [79]
Terminology	Pre-emptive		Clinically driven diagnostic approach		Empirical therapy applied to select pts		Early treatment strategy		Enhance diagnosis with targeted diagnostic testing
Study type	Prospective, feasibility		Prospective, feasibility		Prospective		Retrospective chart review		Prospective care pathway
Pt population	>16 years old and had received chemotherapy for AL or MDS with expected ANC <0.5 × 10^9^ cells/l for at least 10 days or underwent myeloablative allogeneic HSCT		>18 years old with haematological malignancy who underwent chemotherapy or autologous HSCT and developed neutropaenia for at least 7 days		≥16 years old with haematological or solid cancer with persistent febrile neutropaenia (refractory to 5 days of antibiotic treatment and no aetiological diagnosis) post-chemotherapy or after myeloablative HSCT		Pts receiving allogeneic HSCT between 2006 and 2007		Haematology pts admitted at high risk of IFD and entered into the neutropaenic fever management care pathway High risk = SCT, AL, refractory disease with aggressive chemotherapy
Antifungal prophylaxis	100 % fluconazole		None except for secondary prophylaxis with voriconazole		Allogeneic HSCT only—13.5 % Fluconazole		100 % itraconazole unless earlier history of suspected fungal infection, then voriconazole		100 % Itraconazole (SCT/AML) Fluconazole (ALL/lymphoma) Itraconazole (refractory)
IFI diagnostic trigger	Triggered if neutropaenic fever after 5 days of antibiotics or relapsing fever after 48 h of defervescence, clinical signs or symptoms of IFI, appearance of new pulmonary infiltrate while on antibiotics or steroids, isolation of moulds or hyphae on respiratory specimens or two consecutive GM assays ≥0.5		BDWU prior to start of antibiotics If fever after 4 days of antibiotics, relapsing fever after 48 h or febrile pts with evidence of IFI IDWU		Persistent febrile neutropaenia: CXR, blood cultures, CT if abnormal CXR or respiratory symptoms, bronchoscopy in pts with pulmonary infiltrates, abdominal ultrasound if abdominal pain		Neutropaenic and antibiotic-resistant fever at 72 h		Neutropaenic fever included initial workup with blood cultures, urine, and fungal PCR and antigen Antibiotics started and no source identified, blood cultures repeated daily
Diagnostic workup	HRCT with or without sinus CT and bronchoscopy with BAL if no severe hypoxia		BDWU—blood cultures, microbiological, radiological exams IDWU—blood cultures, GM × 3 days, chest CT, microbiological, clinical exams		If severe sepsis/shock or foci of infection not identified, then further workup with CT, abdominal ultrasound and repeat blood cultures		HRCT within 24 h of ongoing fever		No response at 48 h: fungal PCR and antigen twice weekly, consider HRCT scan, viral cultures, CRP
Treatment initiation	Treatment initiated in pts with two consecutive GM assays ≥0.5 or CT findings suggestive of IFI with supportive positive microscopy or culture positive for moulds		Treatment if positive workup or empiric treatment if diagnostic workup negative and pt with persistent neutropaenic fever and worsening clinical conditions		Treatment if severe sepsis/septic shock or foci of infection in lung, CNS, sinus, abdomen or skin Treatment in high-risk pts at the discretion of the provider if negative workup		Treatment if neutropaenic fever and positive HRCT, if HRCT could not be performed within 24 h of ongoing fever or at the discretion of the physician if pt developed respiratory failure with high dependency or ICU, regardless of CT		Treatment given if clinical, microbiological or radiological evidence of IFI Clinical evidence included new cough with pleuritic chest pain, haemoptysis or nodular skin rash or radiological evidence
Treatment regimen	L-AMB		Not specified per protocol—voriconazole, L-AMB, C-AMB, caspofungin and fluconazole reported		L-AMB, C-AMB or voriconazole		Caspofungin		Caspofungin, L-AMB or voriconazole
Outcomes									
Number of pts evaluated	• 88 pts • 136 treatment episodes • 117 episodes of neutropaenic fever		• 146 pts • 220 neutropaenic episodes • 159 episodes with fever		• 66 pts with persistent febrile neutropaenia		• 99 pts • 89 pts with neutropaenic fever • 53 pts with neutropaenic fever at 72 h		• 125 pts with neutropaenic fever
Group	Pre-emptive EIA+	No treatment			EAT	No treatment	Pre-emptive	No treatment	
Number of pts per group	17 (19 episodes)	71 (117 episodes)	48 of 220 episodes received treatment		26	40	17	36	Not reported
Neutropaenia duration <500 cells/μl	Median 19 (4–86)		Median in febrile pts 17 (7–75)		Median 17 (8–37)	Median 12 (6–40)	17 (12–60) Not reported if mean or median		Not reported
Underlying disease	• AML: 42 % • ALL: 19.3 % • MDS: 3.4 % • Relapse AL/MDS: 26.1 % • Other: 9 %		• AML: 45.9 % • ALL: 10.3 % • NHL: 24.7 % • HL: 4.8 % • MM: 13 % • Other: 1.4 %		Neoplasm • Lymphoma: 11.5 % • AL: 73 % • MDS: 7.7 % • SCT: 31 % • Auto: 11.5 % • Allo: 19 %	Neoplasm • Lymphoma: 37.5 %* • AL: 37.5 %** • MDS: 10 % • SCT: 42.5 % • Auto: 32.5 %*** • Allo: 10 %	• AML: 33 % • ALL: 14 % • MDS: 6 % • MM: 7 % • CLL: 11 % • HD: 7 % • CML: 7 % • NHL: 5 % • Other: 9 %		• HSCT: 44 % • AML: 31 % • ALL: 0.8 % • CLL: 3.2 % • CML: 0.8 % • NHL: 18 % • HD: 0.8 % • AA: 1.6 %
Indication for treatment	• Positive GM: 16 episodes • Persistent fever: 3 episodes	None	• Treatment all diagnostic-driven, except in one pt who received treatment for persistent febrile neutropaenia and worsening clinical symptoms		• Septic shock: 34.6 % • Fever with defined focus: 30.7 % • Clinical decision: 34.6 %	None	• Positive CT: 15 pts • Empirical until CT performed and was negative: 2 pts	None	
Reported organisms	BAL: *Aspergillus* spp. 31.6 % (6 pts) NFP 57.9 % (11 pts) Not done 10.59 % (2 pts)	2 cases breakthrough *C*. *glabrata* 1 case disseminated zygomycosis	Possible IFD—16 cases Proven/probable IA—27 cases Proven/probable IZ—3 cases Candidaemia—3 cases		Proven/probable IFD (3 pts): *Aspergillus* spp.—67 % *Scedosporium prolificans*—33 %	None	*A*. *fumigates*: 1 pt	None	*Aspergillosis* PCR + GM-EIA positive—25 pts PCR positive—36 pts GM-EIA positive—7 pts *Candidosis* PCR + M-EIA positive: 9 pts PCR positive: 2 pts M-EIA positive: 1 pt Negative all tests: 55 pts
Overall mortality	16/88 (18.1 %)		36/146 (24.6 %)		8/26 (31 %)	2/40 (5 %)	2/17 (11.7 %^a^)	3/36 (8.3 %^a^)	42/125 (33.6 %)
IFD mortality	Primary—2/17 (11.7 %^a^) Contributing—4/17 (24 %^a^) EIA positive—7/17 (41.1 %)		Primary cause in IFD group—4/36 (11.1 %) Primary cause overall—4/146 (2.7 %)		2/26 (8 %)	0	1 (5.9 %^a^)	0	10/125 (8 %)
Time frame	12 weeks		3 months		30 days		100 days		1 year

*AA* aplastic anaemia, *AL* acute leukaemia, *ALL* acute lymphocytic leukaemia, *AML* acute myelogenous leukaemia, *ANC* absolute neutrophil count, *BAL* bronchoalveolar lavage, *BDWU* basic diagnostic workup, *C-AMB* conventional amphotericin B, *CLL* chronic lymphocytic leukaemia, *CML* chronic myeloid leukaemia, *CNS* central nervous system, *CRP* C-reactive protein, *CT* computed tomography, *CXR* chest X-ray, *EAT* early antifungal therapy, *EIA* enzyme immunoassay, *GM* galactomannan, *GVHD* graft-versus-host disease, *HD* Hodgkin’s disease, *HL* Hodgkin’s lymphoma, *HRCT* high-resolution computed tomography, *HSCT* haematopoietic stem cell transplant, *IA* invasive aspergillosis, *ICU* intensive care unit, *IDWU* intensive diagnostic workup, *IFD* invasive fungal disease, *IFI* invasive fungal infection, *IZ* invasive zygomycosis, *L-AMB* liposomal amphotericin B, *MDS* myelodysplastic syndrome, *M-EIA* mannan enzyme immunoassay, *MM* multiple myeloma, *NFP* no fungal pathogen, *NHL* non-Hodgkin’s lymphoma, *PCR* polymerase chain reaction, *Pt* patient, *SCT* stem cell transplant

^a^Percentage calculated from the reported number of pt deaths

*p = 0.02, **p = 0.005, ***p = 0.04

**Table 3:** Summary of randomised, controlled trials comparing pre-emptive and empirical treatments

Country	France (Cordonnier et al. 2009) [25]		Germany (Hebart et al. 2009) [39]	
Pt population	≥18 years with haematological malignancy scheduled for chemotherapy or autologous HSCT and expected to cause neutropaenia for at least 10 days		Allogeneic HSCT	
Antifungal prophylaxis	Site-specific (range 42–48 %)		100 % oral amphotericin B, fluconazole	
Treatment	Empirical	Pre-emptive	Empiric	PCR-based
	• Therapy started on Day 4 of persistent fever with antibiotic treatment • Recurrent fever between Days 4 and 14	After 4 days of fever and antibiotic treatment • Clinically and imaging documented pneumonia or acute sinusitis • Grade 3 mucositis • Septic shock • Skin lesion suggesting IFD • Unexplained CNS symptoms • Periorbital inflammation • Splenic or hepatic abscess • Severe diarrhoea • *Aspergillus* colonisation • Positive GM defined as an index ≥1.5	• Febrile neutropaenia >120 h not responsive to broad-spectrum antibiotics • Detection of pulmonary infiltrate	• One positive PCR result • Febrile neutropaenia >120 h not responsive to broad-spectrum antibiotics • Detection of pulmonary infiltrate
Treatment regimen	L-AMB or C-AMB depending on renal function		L-AMB	
Outcomes	Empirical	Pre-emptive	Empirical	PCR-based
Pts randomised	150	143	207	196
Pts receiving antifungal treatment	92	56	76	112
Duration of neutropaenia <500 cells/mm^3^ (days)	Median 18 (6–69)	Median 17 (5–57)	Not reported	Not reported
Indication for treatment	• Isolated fever 4–14 days after antibiotics—59.8 % • Pneumonia—6.5 % • Severe mucositis—8.7 % • Isolated fever beyond Day 14—12.0 % • Septic shock—5.4 % • +GM test—2.2 % • Skin lesion—2.2 % • Sinusitis or periorbital inflammation—0 • Neurological symptoms—2.2 % • Diarrhoea—1.1 %	• Isolated fever 4–14 days after antibiotics—1.8 %** • Pneumonia—46.4 % • Severe mucositis—17.9 % • Isolated fever beyond Day 14—12.5 % • Septic shock—5.4 % • +GM test—5.4 % • Skin lesion—3.6 % • Sinusitis or periorbital inflammation—5.4 % • Neurological symptoms—0 • Diarrhoea—1.8 %	Most frequently reported • Fever—58 % • Pulmonary infiltrates—22.6 %	Most frequently reported • PCR-based—49.7 % • Fever—27.2 % • Pulmonary infiltrates—12.4 %
Antifungal use	61.3 %	39.2 %**	36.7 %	57.1 %***
IFD	2.7 %	9.1 %*	8.2 %	8.2 %
Reported organisms	Proven/probable—4 cases *Aspergillus* spp.—100 %	Proven/probable—13 cases *Aspergillus* spp.—61.5 % *Candida* spp.—38.5 %	Proven IFDs: 16 cases *Candida*—69 % *Aspergillus*—25 % *Candida* and *Aspergillus*—6 %	Proven IFDs: 12 cases *Candida*—33.3 % *Aspergillus*—33.3 % *Candida* and *Aspergillus*—33.3 %
Mortality	4/150 (2.7 %)	7/143 (4.9 %)	30 days: 6.3 % 91 days: 16.4 %	30 days: 1.5 %^a^ 91 days: 16.3 %
IFI IFD mortality	0	3/143 (2.1 %)	10/76 (13.2 %)	7/112 (6.3 %)
Time frame	14 days after recovery from neutropaenia or if persistent neutropaenia 60 days after study inclusion or adverse event		30 days, 91 days (12-week mortality benchmark)	

*C-AMB* conventional amphotericin B, *CNS* central nervous system, *GM* galactomannan, *HSCT* haematopoietic stem cell transplant, *IFD* invasive fungal disease, *L-AMB* liposomal amphotericin B, *PCR* polymerase chain reaction, *Pt*, patient

*p < 0.02, **p < 0.001, ***p < 0.0001, ^a^ *p* = 0.015

In the comparative studies, the incidence of IFDs ranged between 2.7 and 8.2 % in empirically treated patients and between 8.2 and 9.1 % in the pre-emptively treated patients [25, 39]. In the study conducted by Cordonnier and colleagues, the difference in the IFD rate between the treatment groups was statistically significant (pre-emptive 9.1 % vs. empirical 2.7 %, *p* < 0.02) [25]. The rate of IFDs was significantly higher in a subgroup of patients who received induction therapy (16.4 %) versus patients who received consolidation therapy/autologous stem cell transplantation (3.9 %, *p* < 0.01), with 15/17 IFD cases occurring in the induction group and most infections occurring in the pre-emptive arm (12 cases of IFD). Despite the randomised study design, there were differences between the two treatment groups, which must be taken into account when interpreting these data. For example, greater morbidity was recorded for patients in the pre-emptive group versus the empirical group. Outcomes were similar between pre-emptive and empirical strategies in the study by Hebart and colleagues, where patient demographics were more consistent, all patients were on prophylaxis at baseline and only liposomal amphotericin B was used [39].

The overall mortality in the two comparative trials ranged from 1.5 to 16.5 % and IFD mortality ranged from 0 to 13.2 % [25, 39]. The study by Cordonnier and colleagues demonstrated non-inferiority in the overall population between the empirical and pre-emptive groups with regards to survival at 2 weeks after recovery from neutropaenia [25]. In a subgroup analysis of patients who received induction therapy, the inferiority of pre-emptive versus empirical treatment in terms of 2-week survival could not be ruled out, but the study was not powered to prove this. This may be related to a longer median duration of neutropaenia in the pre-emptive versus the empirical arm in patients receiving induction therapy (26 days vs. 12 days, respectively). By contrast, non-inferiority was demonstrated for empirical versus pre-emptive treatment in a subgroup of patients who received consolidation therapy/autologous stem cell transplantation. While empirical treatment may be comparatively beneficial versus pre-emptive treatment in patients receiving induction therapy (but not in patients receiving consolidation therapy), the differences between the groups in this study must be considered.

In the Hebart study, mortality at Day 100 did not differ between the pre-emptive and empirical groups, although the pre-emptive group had better survival at Day 30 [39]. Pre-emptive treatment was initiated after a positive PCR test; after Day 30, regular PCR testing became more difficult, as most patients had been discharged from hospital. A study with consistent PCR testing beyond 30 days would be instructive in delineating whether pre-emptive treatment confers a tangible survival benefit over empirical therapy.

The studies reviewed herein indicated that IFD cases may be missed less frequently when patients are treated pre-emptively versus empirically. In total, seven IFD cases were missed in three studies. Four proven cases of IFD were missed in the empirical group and one proven case of IFD was missed in the PCR-triggered pre-emptive group reported by Hebart et al. [39]. Maertens and colleagues identified one case of invasive zygomycosis where a patient in the empirical group did not receive antifungal treatment [17]. This patient did not present with fever or other signs of IFDs. By contrast, ten patients were treated pre-emptively due to positive galactomannan testing, despite being non-febrile or having another source of fever identified. These patients had no evidence of IFDs and would not have received treatment via an empirical fever-driven strategy.

Cordonnier and colleagues defined breakthrough infections as infections documented ≥24 h after the first dose of antifungal treatment. The occurrence of breakthrough infections was similar for the empirical and pre-emptive treatment arms (empirical arm *Aspergillus* infections, 1.3 % of patients; pre-emptive arm *Aspergillus* species or *Candida* species, 1.4 % of patients for both organisms) [25]. Two cases of breakthrough candidaemia with *C*. *glabrata* were identified with blood cultures in a study of pre-emptive treatment by Maertens and colleagues [17].

Overall, excluding the study by Hebart et al. [39], empirical or potentially empirical antifungal use ranged from 35 to 61.3 %, while pre-emptive antifungal use ranged from 7.7 to 39.4 %. Thus, five out of six studies demonstrated an overall decrease in antifungal use by 43–78 % with the use of a pre-emptive treatment versus empirical strategy [17, 25, 51, 74, 79, 82].

Limited data are available comparing the cost-effectiveness of pre-emptive versus empirical strategies for IFDs. However, a benefit in terms of cost and LOS in high-risk patients has been suggested for pre-emptive treatment [79].

These results emphasise the diverse diagnostic methods and therapeutic modalities that can be used within a pre-emptive strategy. Various IFDs were represented in the studies, including *Candida* species. However, when patient populations were consistent, all patients received prophylaxis at baseline and amphotericin B was used by all patients, and outcomes were similar for pre-emptive and empirical treatment strategies [39]. The rates of breakthrough infection were comparable. The rates of antifungal use were lower with pre-emptive treatment and infection may be detected more frequently using this strategy. Additional studies of pre-emptive antifungal treatment are warranted.

### Economic burden of IFDs

Fifteen studies across eight countries reported economic or HCRU data; these studies reported greater costs in patients with IFDs versus patients without infection. In an observational study in patients with acute myelogenous leukaemia (AML)–myelodysplastic syndromes (MDS) receiving high-dose chemotherapy, the mean total cost per patient was €57,750 with no IA, €68,280 with possible IA and €83,300 with probable or proven IA [61]. The additional IA cost burden ranged from €10,530 to €25,550, and was statistically significantly greater across all areas of expenditure in patients with possible, probable or proven IA versus patients without IA (*p* < 0.001) [61]. A longer LOS was noted for patients with possible IA (91 days) and probable or proven IA (104 days) versus patients without IA (84 days) [61].

Gangneux and colleagues reported on 50 patients with AML with probable or proven IFD followed for 1 year [29]. LOS for index hospitalisation was 45 days (82 % of stays were for malignancy treatment) and the mean duration of antifungal treatment was 198 days [29]. The increase in treatment costs for an IFD episode was €51,033; antifungals accounted for €35,967 (70.5 %) of this expenditure during the year [29]. The cost of index hospitalisation was €13,721, plus €1,345 for each additional hospitalisation [29]. Berger and colleagues reported on the burden of IFDs in patients who had undergone remission induction chemotherapy for AML or MDS and included those with proven or probable IFD receiving antifungal treatment [35]. Overall, patients with IFDs stayed in hospital 12 days longer than patients without IFDs [35].

The economic impact of IFDs can be assessed from the hospital, payer or societal perspectives. Although the hospital perspective includes only costs that are incurred by the hospital (e.g. costs of diagnostic tests, medications, hospitalisation), the payer perspective includes all direct medical costs (e.g. primary treatment costs, costs of HCRU post-discharge) and the societal perspective includes all direct medical and non-medical costs (e.g. lost productivity), as well as indirect costs (e.g. future lost productivity).

#### Hospital perspective

The hospital perspective costs varied depending on the resources included in the analysis. Costs generally ranged from €8,351 to €11,821 when evaluating incremental hospitalisation and antifungal drug expenditure only, €3,930–€7,314 for antifungal drugs, €8,252–€51,760 for hospital bed day costs and €26,596–€83,300 when all direct costs for management were included [35–37, 58, 59, 61, 67, 68, 77, 86].

#### Payer perspective

Bruynesteyn and colleagues used a decision tree to compare the cost-effectiveness of caspofungin versus liposomal amphotericin B from the UK National Health Service perspective [80]. The average direct treatment costs were £9,763 for caspofungin and £11,795 for liposomal amphotericin B, where the drug costs were £4,601 and £6,395, respectively [80]. The difference in other direct costs, including hospitalisation and drug costs related to the management of adverse events, was £239 in favour of caspofungin up to a weight of 77 kg [80]. Van Campenhout and colleagues compared cost data from an observational study with a model utilising voriconazole for IA therapy [19]. The average total costs of treatment when considering only hospital days associated with fungal infection was €12,376, and most of this was associated with antifungal use [19].

#### Societal perspective

Two studies reported costs from the societal perspective, although one study reported a narrow societal perspective and only included direct costs [40, 60]. Both studies used a Markov model and compared voriconazole with conventional amphotericin B for IA, with one study also including itraconazole [40, 60]. The mean treatment costs for voriconazole and conventional amphotericin B ranged from €25,353 to €26,974 in a 12-week model and €30,026–€33,616 in a life-long model [40, 60].

## Summary and discussion

Despite the availability of a range of therapeutic options, the clinical and economic burden of IFDs remains high.

Considerable variation in reporting methods for IFD-related mortality data was observed, making interpretation difficult. In many cases, denominator data were not included and attributable mortality could not be differentiated from crude mortality. Some studies may have been affected by ascertainment bias [17, 25, 63, 78]. The inclusion of some biomarkers (e.g. galactomannan, β-D-glucan) and not others (e.g. PCR), and the heavy reliance on specific radiological signs in EORTC/MSG criteria, has led to a stricter definition of IFD, resulting in a significant reduction of possible/probable IFD cases, with an anticipated improvement in specificity for clinical trials [2], However, the criteria are unsuitable for the evaluation of new diagnostic tools because existing criteria are used to define the disease, which may lead to ascertainment bias.

Guidelines provide recommendations for the use of biological tests in adult patients [91, 92]. Although strong evidence supports the use of galactomannan testing in serum, only moderate evidence supports the use of combined mannan/anti-mannan testing in serum [91]. Anti-mannan antibody testing was not included in this analysis. No PCR recommendations were proposed, owing to a lack of standardisation [91]. HRCT scans and the galactomannan assay were used for diagnosis, although cut-offs and the frequency of testing varied. Utilisation of the (1,3)-β-d-glucan assay and PCR testing was limited.

A variety of terms were used to describe strategies for the prevention and treatment of IFD, leading to confusion and overlap. Antifungal prophylaxis was used in many studies, but the definition of efficacy or failure varied. Empirical treatment was generally defined as the initiation of antifungal therapy in a neutropaenic patient with persistent fever, despite broad-spectrum antibiotics for 4 to 7 days. Other terms included universal empirical and early antifungal therapy. Several studies misinterpreted pre-emptive therapy, a strategy which aims to detect infection before clinical disease develops. When clinical and radiological disease is present, the opportunity for the pre-emptive therapy of infection has passed. Other terms identified included clinically driven, diagnostic-driven treatment and targeted prophylaxis/therapy.

Although there is a dearth of studies comparing empirical and pre-emptive treatment strategies, only two randomised, controlled studies were identified. The variations in diagnosis and treatment in these studies led to difficulties in developing conclusions regarding the role of pre-emptive therapy. A prospective, randomised clinical study comparing empirical and diagnostic-driven (pre-emptive) therapy in patients with acute leukaemia or undergoing allogeneic SCT is ongoing in European centres (NCT01288378).

Studies assessing the economic burden of IFDs are limited; the incremental cost burden is estimated to be between €10,530 and €51,033, depending on the certainty of infection and the duration of follow-up. Drivers of HCRU burden included hospitalisation, diagnostic testing and medications. To demonstrate a clear cost benefit, further evidence that a pre-emptive strategy decreases antifungal use compared with a standard empirical approach is needed.

In conclusion, the primary evidence reported here demonstrates the importance of IFDs in Europe and highlights the need for early and appropriate therapy, facilitated by new techniques for more rapid diagnosis. Additional studies are required in order to establish the clinical and economic burden of IFDs, and assess current treatment practices.

## Electronic supplementary material

Below is the link to the electronic supplementary material.

ESM 1 (DOC 101 kb)
