# Supplementary material for: Clinical and economic burden of invasive fungal diseases in Europe: focus on pre-emptive and empirical treatment of Aspergillus and Candida species
Source: Eur J Clin Microbiol Infect Dis. 2013 Sep 12;33(1):7–21. doi: 10.1007/s10096-013-1944-3 (PMC3892112; doi:10.1007/s10096-013-1944-3)
Supplement: Supplementary file 1 — (DOC 101 kb) [file 10096_2013_1944_MOESM1_ESM.doc]

# Journal: *European Journal of Clinical Microbiology & Infectious Diseases*

# Title: Clinical and economic burden of invasive fungal diseases in Europe: focus on pre-emptive and empirical treatment of *Aspergillus* and *Candida* species

**Authors:** L. Drgona, A. Khachatryan, J. Stephens, C. Charbonneau, M. Kantecki, S. Haider, R. Barnes

**Corresponding author:** Jennifer Stephens, Pharmerit International, E-mail: jstephens@pharmerit.com

**Supplementary material**

*Study eligibility and selection criteria*

Bibliographies from full-text articles were reviewed and cross-referenced to the master database. Where this cross-reference process yielded an article that was missed in the electronic search, the citation was added to the master reference database and reviewed. Following application of the inclusion and exclusion criteria, a final collection of articles were eligible for data extraction and further synthesis. The synthesis and reporting of results are descriptive in nature. No statistical techniques were used to combine studies and results represent raw data only.

*Risk factors for mortality*

Risk factors for mortality in patients infected with *Candida* species included: age >50 years or ≥65 years [1–3]; shock [2, 3]; acute or chronic renal failure [1, 2]; gastrointestinal dysfunction; recent or concurrent bacteraemia; or endotracheal intubation [1]. Infection with *C.* non-*albicans* was reported as a risk factor when compared to infection with *C. albicans* [4].

For invasive aspergillosis (IA), allogeneic haematopoietic stem cell transplant patients had a greater risk of mortality and predictors of death included: age >36 years; the presence of a central vein catheter; and receipt of transplant from a matched, unrelated donor [5]. Gallien and colleagues reported an association between neutropenia and an unsuccessful outcome, and a higher risk of death was reported for patients with acute myeloid leukaemia with IA in cases of remission or failure of chemotherapy [6].

Severe renal insufficiency at baseline, defined as a creatinine clearance rate of <30 mL/min, significantly increased the risk of death (p=0.004) at 12 weeks in patients with invasive fungal diseases (IFD) [7]. Hahn-Ast and colleagues reported factors predictive for overall survival in neutropenic patients with IFD post-myelosuppressive chemotherapy, including: controlled disease after chemotherapy; possible IFD; age <60 years; the 2002–2006 time period; and the use of a novel antifungal treatment consisting of caspofungin and/or voriconazole [8]. A better defined set of risk factors for mortality due to IFD may assist in prioritising patients who could receive greatest benefit from early antifungal treatment.

**Supplementary Table 1** Search terminology

| **CDI subject area** | **Search terms** |
| --- | --- |
| **Parent literature collection** | **Invasive fungal infection**  *“Invasive fungal infection”*  *“Invasive, fungal, infection, fungal infection”*  *“Mycoses”* (in “All fields”, MeSH and EMTREE headings) |
|  | **Europe**  *“Europe”* (in “All fields”, MeSH and EMTREE headings) |
|  | **Organisms**  *“Aspergillosis, Aspergillus”* (in “All fields”, MeSH and EMTREE headings)  *“Candidiasis, Candida”* (in “All fields”, MeSH and EMTREE headings) |
| **Clinical burden** | **Epidemiology**  *“Epidemiology”, “prevalence”, “incidence”, “epidemiologic methods”* (MeSH) |
|  | **Clinical outcomes**  *“Mortality, survival, survival rate, death”* (in “All fields”, MeSH and EMTREE headings) |
|  | **Morbidity**  *“Morbidity”* (in “All fields”, MeSH and EMTREE headings) |
|  | **Diagnosis**  *“Diagnosis”* (in “All fields”, MeSH and EMTREE headings) |
|  | **Treatment**  *“Treatment, therapy, outcome”*  *“Therapeutics”* (in “All fields”, MeSH and EMTREE headings) |
| **Economic burden** | **Resource utilisation**  *“Resource utilisation”*  *“Cost burden”*  *“Economic burden”* |
|  | **Economic analyses**  *“Cost”*  *“Cost of illness, healthcare costs, cost benefit analysis”* (MeSH and EMTREE headings)  *“Economics”* (in “All fields” MeSH and EMTREE headings) |

CDI: Clinical Documentation Improvement; EMTREE: Elsevier's Life Sciences Thesaurus; MeSH, Medical Subject Headings

**Supplementary Table 2** Inclusion and exclusion criteria

|  | **Inclusion criteria** | **Exclusion criteria** |
| --- | --- | --- |
| **Abstract eligibility criteria** | - Contains any derivative of IFD related to *Aspergillus* or *Candida* species - Results in English - Published in last 10 years - Studies in humans | - No abstract available - Pertains to colonisation or prophylaxis - No infection, infection unrelated to IFD or *Aspergillu*s/*Candida* species - Specific mention of data derived from patients outside Europe - Case studies |
| **Full-text eligibility and selection criteria** | - IFD with *Aspergillus* or *Candida* species - No restriction on interventions - No restriction on comparisons - Clinical (mortality, treatment timing etc) - Economic (cost, length of stay etc) - Patient, payer, hospital or societal perspective | - Non-English language - Case studies - Commentary - Sample size < 20 patients - Non-invasive infection - Infections other than IFD or *Aspergillus*/*Candida* species - No costs or clinical data in results related to IFD - *In vitro* results only - Country of origin for results cannot be verified |

IFD: invasive fungal disease

**Supplementary Table 3** Rates and risk factors for mortality by country

| **Country** | **Overall mortality (%)** | **IFD-related mortality (%)** | **Factors associated with IFD mortality** |
| --- | --- | --- | --- |
| Austria [9, 10] | 33.2–34 | 77 |  |
| Belgium [11–13] | 14–86 | 6 |  |
| Finland [14] |  | 24 |  |
| France [6, 7, 15–18] | 27–73 | 0–52 | - Chemotherapy failure - Immunosuppression - Mechanical ventilation - Neutropenia - Remission - Renal insufficiency - Type-1 diabetes |
| Germany [8, 19] | 1.5–36 | 6.3–57.4 |  |
| Greece [4, 20] | 11.1–90 |  |  |
| Ireland [1, 21] | 20.6–39.7 |  | - Acute renal failure - Age ≥65 years - Endotracheal intubation - Recent/concurrent bacteremia - Severe gastrointestinal dysfunction |
| Italy [5, 22–26] | 24.1–38.2 | 0–65.3 | - Allogeneic HSCT - AML stage - Age >36 years - HSCT cell source - Presence of central vein catheter - Recovery from neutropenia |
| The Netherlands [27] | 22 | 10 |  |
| Slovakia [28, 29] | 33.7 | 21 |  |
| Spain [2, 3, 30–32] | 20.2–90 | 22.8–55 | - Age - Chronic renal failure - Shock |
| Switzerland [33] | 30 | 35–81 |  |
| UK [34, 35] | 5–59.6 | 8–13 |  |
| Europe [36, 37] | 32–58 | 45 |  |

AML acute myeloid leukaemia; HSCT: haematopoietic stem cell transplant; IFD: invasive fungal diseases

**References**

1. Boo TW, O'reilly B, O'leary J, Cryan B (2005) Candidaemia in an Irish tertiary referral hospital: epidemiology and prognostic factors. Mycoses 48:251–259

2. Fortun J, Martin-Davila P, Ros L, Luengo J, Agundez M, Sanchez-Sousa A, et al (2009) Impact of epidemiological changes in candidemia on outcome. Abstract presented at the Interscience Conference on Antimicrobial Agents and Chemotherapy, September 12-14, San Francisco, CA, USA

3. Ortega M, Alemla M, Soriano A, Marco F, Martinez J, Pitart C, Nicolau C, Mensa J (2010) Analysis of 540 episodes of Candida bloodstream infection isolated in a single institution from Barcelona: impact of treatment in outcome. Abstract presented at the Interscience Conference on Antimicrobial Agents and Chemotherapy, September 12-15, Boston, MA, USA

4. Dimopoulos G, Ntziora F, Rachiotis G, Armaganidis A, Falagas ME (2008) *Candida albicans* versus non-albicans intensive care unit-acquired bloodstream infections: differences in risk factors and outcome. Anesth Analg 106:523–529

5. Pagano L, Caira M, Nosari A, Van Lint MT, Candoni A, Offidani M, Aloisi T, Irrera G, Bonini A, Picardi M, Caramatti C, Invernizzi R, Mattei D, Melillo L, de Waure C, Reddiconto G, Fianchi L, Valentini CG, Girmenia C, Leone G et al (2007) Fungal infections in recipients of hematopoietic stem cell transplants: results of the SEIFEM B-2004 study--Sorveglianza Epidemiologica Infezioni Fungine Nelle Emopatie Maligne. Clin Infect Dis 45:1161–1170

6. Gallien S, Fournier S, Porcher R, Bottero J, Ribaud P, Sulahian A, Socié G, Molina JM (2008) Therapeutic outcome and prognostic factors of invasive aspergillosis in an infectious disease department: a review of 34 cases. Infection 36:533–538

7. Lortholary O, Charlemagne A, Bastides F, Chevalier P, Datry A, Gonzalves MF, Michel G, Tilleul P, Veber B, Herbrecht R (2004) A multicentre pharmacoepidemiological study of therapeutic practices in invasive fungal infections in France during 1998-1999. J Antimicrob Chemother 54:456–464

8. Hahn-Ast C, Glasmacher A, Mückter S, Schmitz A, Kraemer A, Marklein G, Brossart P, von Lilienfeld-Toal M (2010) Overall survival and fungal infection-related mortality in patients with invasive fungal infection and neutropenia after myelosuppressive chemotherapy in a tertiary care centre from 1995 to 2006. J Antimicrob Chemother 65:761–768

9. Presterl E, Daxböck F, Graninger W, Willinger B (2007) Changing pattern of candidaemia 2001-2006 and use of antifungal therapy at the University Hospital of Vienna, Austria. Clin Microbiol Infect 13:1072–1076

10. Perkhofer S, Lass-Flörl C, Hell M, Russ G, Krause R, Hönigl M, Geltner C, Auberger J, Gastl G, Mitterbauer M, Willinger B, Knöbl P, Resch G, Waldner R, Makrai A, Hartmann G, Girschikofsky M, Greil R (2010) The Nationwide Austrian Aspergillus Registry: a prospective data collection on epidemiology, therapy and outcome of invasive mould infections in immunocompromised and/or immunosuppressed patients. Int J Antimicrob Agents 36:531–536

11. Van Campenhout H, Marbaix S, Derde MP, Annemans L (2008) Voriconazole treatment of invasive aspergillosis: real-world versus health-economic model results. Clin Drug Investig 28:509–521

12. Vandewoude K, Blot S, Benoit D, Depuydt P, Vogelaers D, Colardyn F (2004) Invasive aspergillosis in critically ill patients: analysis of risk factors for acquisition and mortality. Acta Clin Belg 59:251–257

13. Maertens J, Theunissen K, Verhoef G, Verschakelen J, Lagrou K, Verbeken E, Wilmer A, Verhaegen J, Boogaerts M, Van Eldere J (2005) Galactomannan and computed tomography-based preemptive antifungal therapy in neutropenic patients at high risk for invasive fungal infection: a prospective feasibility study. Clin Infect Dis 41:1242–1250

14. Anttila VJ, Salonen J, Ylipalosaari P, Koivula I, Riikonen P, Nikoskelainen J (2007) A retrospective nationwide case study on the use of a new antifungal agent: patients treated with caspofungin during 2001-2004 in Finland. Clin Microbiol Infect 13:606–612

15. Cordonnier C, Ribaud P, Herbrecht R, Milpied N, Valteau-Couanet D, Morgan C, Wade A (2006) Prognostic factors for death due to invasive aspergillosis after hematopoietic stem cell transplantation: a 1-year retrospective study of consecutive patients at French transplantation centers. Clin Infect Dis 42:955–963

16. Herbrecht R, Cordonnier C, Gangneux J, Caillot D, Auvrignon A, Thiebaut A, Gorin N, Brethon B, Michallet M., Fisher A, Bertrand Y, Preziosi P, Ruiz F (2010) Management and outcomes of invasive fungal diseases (IFD) in patients with hematological malignancies: a French Cohort study. Abstract presented at the Interscience Conference on Antimicrobial Agents and Chemotherapy, September 12-15, Boston, MA, USA

17. Fourneret-Vivier A, Lebeau B, Mallaret MR, Brenier-Pinchart MP, Brion JP, Pinel C, Garban F, Pison C, Hamidfar R, Plantaz D, Pelloux H, Grillot R (2006) Hospital-wide prospective mandatory surveillance of invasive aspergillosis in a French teaching hospital (2000-2002). J Hosp Infect 62:22–28

18. Cordonnier C, Pautas C, Maury S, Vekhoff A, Farhat H, Suarez F, Dhédin N, Isnard F, Ades L, Kuhnowski F, Foulet F, Kuentz M, Maison P, Bretagne S, Schwarzinger M (2009) Empirical versus preemptive antifungal therapy for high-risk, febrile, neutropenic patients: a randomized, controlled trial. Clin Infect Dis 48:1042–1051

19. Hebart H, Klingspor L, Klingebiel T, Loeffler J, Tollemar J, Ljungman P, Wandt H, Schaefer-Eckart K, Dornbusch HJ, Meisner C, Engel C, Stenger N, Mayer T, Ringden O, Einsele H (2009) A prospective randomized controlled trial comparing PCR-based and empirical treatment with liposomal amphotericin B in patients after allo-SCT. Bone Marrow Transplant 43:553–561

20. Roilides E, Farmaki E, Evdoridou J, Dotis J, Hatziioannidis E, Tsivitanidou M, Bibashi E, Filioti I, Sofianou D, Gil-Lamaignere C, Mueller FM, Kremenopoulos G (2004) Neonatal candidiasis: analysis of epidemiology, drug susceptibility, and molecular typing of causative isolates. Eur J Clin Microbiol Infect Dis 23:745–750

21. Scanlon N, Jaura A, Fraher M, Collins C, Marsh B, McWade R, Hannan M (2008) A Change in Epidemiology of Fungal Infections in ICU Patients: The Impact of Antifungal Therapy. Abstract presented at the Interscience Conference on Antimicrobial Agents and Chemotherapy/Infectious Diseases Society of America, October 25-28, Washington, DC, USA

22. Lequaglie C (2002) Liposomal amphotericin B (AmBisome): efficacy and safety of low-dose therapy in pulmonary fungal infections. J Antimicrob Chemother 49 Suppl 1:49–50

23. Girmenia C, Pizzarelli G, Cristini F, Barchiesi F, Spreghini E, Scalise G, Martino P (2006) Candida guilliermondii fungemia in patients with hematologic malignancies. J Clin Microbiol 44:2458–2464

24. Riva M, Nosari A, rizio A, Molteni A, Vanelli C, Gabutti C, Nador G, Morra E (2008) In high risk acute leukemia patients early antifungal therapy (EAT) improves the outcome of invasive mould infections. Abstract presented at the Interscience Conference on Antimicrobial Agents and Chemotherapy/Infectious Diseases Society of America, October 25-28, Washington, DC, USA

25. Girmenia C, Micozzi A, Gentile G, Santilli S, Arleo E, Cardarelli L, Capria S, Minotti C, Cartoni C, Brocchieri S, Guerrisi V, Meloni G, Foà R, Martino P (2010) Clinically driven diagnostic antifungal approach in neutropenic patients: a prospective feasibility study. J Clin Oncol 28:667–674

26. Bedini A, Venturelli C, Mussini C, Guaraldi G, Codeluppi M, Borghi V, Rumpianesi F, Barchiesi F, Esposito R (2006) Epidemiology of candidaemia and antifungal susceptibility patterns in an Italian tertiary-care hospital. Clin Microbiol Infect 12:75–80

27. Slobbe L, Polinder S, Doorduijn JK, Lugtenburg PJ, el Barzouhi A, Steyerberg EW, Rijnders BJ (2008) Outcome and medical costs of patients with invasive aspergillosis and acute myelogenous leukemia-myelodysplastic syndrome treated with intensive chemotherapy: an observational study. Clin Infect Dis 47:1507–1512

28. Drgona L, Trupl J, Hupkova H (2010) Clinical analysis of 92 patients with fungaemia – data from national survey in Slovak. Poster presented at the 14th International Congress on Infectious Diseases, March 9-12, Miami, FL, USA

29. Drgona L, Kotulova D, Trupl J, Kunova A, Hupkova H, Lisalova M, Marcek T (2011) Trends of candidaemia in Slovakia – a comparison of two consecutive, prospective studies. Poster presented at the 21st European Congress of Clinical Microbiology and Infectious Diseases, May 7-10, Milan, Italy

30. Guinea J, Jenson J, Torres-Narbona M, Gijon Pelaez T, Munoz P, Bouza E (2008) Chronic obstructive pulmonary disease (COPD) is currently the most predisposing condition to invasive aspergillosis. Abstract presented at the Interscience Conference on Antimicrobial Agents and Chemotherapy/Infectious Diseases Society of America, October 25-28, Washington, DC, USA

31. Rubio PM, Sevilla J, González-Vicent M, Lassaletta A, Cuenca-Estrella M, Díaz MA, Riesco S, Madero L (2009) Increasing incidence of invasive aspergillosis in pediatric hematology oncology patients over the last decade: a retrospective single centre study. J Pediatr Hematol Oncol 31:642–646

32. Almirante B, Rodríguez D, Cuenca-Estrella M, Almela M, Sanchez F, Ayats J, Alonso-Tarres C, Rodriguez-Tudela JL, Pahissa A (2006) Epidemiology, risk factors, and prognosis of Candida parapsilosis bloodstream infections: case-control population-based surveillance study of patients in Barcelona, Spain, from 2002 to 2003. J Clin Microbiol 44:1681–1685

33. Weisser M, Rausch C, Droll A, Simcock M, Sendi P, Steffen I, Buitrago C, Sonnet S, Gratwohl A, Passweg J, Fluckiger U (2005) Galactomannan does not precede major signs on a pulmonary computerized tomographic scan suggestive of invasive aspergillosis in patients with hematological malignancies. Clin Infect Dis 41:1143–1149

34. Dignan FL, Evans SO, Ethell ME, Shaw BE, Davies FE, Dearden CE, Treleaven JG, Riley UB, Morgan GJ, Potter MN (2009) An early CT-diagnosis-based treatment strategy for invasive fungal infection in allogeneic transplant recipients using caspofungin first line: an effective strategy with low mortality. Bone Marrow Transplant 44:51–56

35. Barnes RA, White PL, Bygrave C, Evans N, Healy B, Kell J (2009) Clinical impact of enhanced diagnosis of invasive fungal disease in high-risk haematology and stem cell transplant patients. J Clin Pathol 62:64–69

36. Lortholary O, Ascioglu S, Moreau P, Herbrecht R, Marinus A, Casassus P, De Pauw B, Denning DW (2000) Invasive aspergillosis as an opportunistic infection in nonallografted patients with multiple myeloma: a European Organization for Research and Treatment of Cancer/Invasive Fungal Infections Cooperative Group and the Intergroupe Français du Myélome. Clin Infect Dis 30:41–46

37. Tortorano AM, Peman J, Bernhardt H, Klingspor L, Kibbler CC, Faure O, Biraghi E, Canton E, Zimmermann K, Seaton S, Grillot R (2004) Epidemiology of candidaemia in Europe: results of 28-month European Confederation of Medical Mycology (ECMM) hospital-based surveillance study. Eur J Clin Microbiol Infect Dis 23:317–322
